# The impact of blood lactic acid levels on retinopathy of prematurity morbidity

**DOI:** 10.1186/s12887-024-04571-y

**Published:** 2024-02-29

**Authors:** Congcong Zhao, Zhihong Sun, Hongming Chen, Kaili Li, Huiqing Sun

**Affiliations:** 1 Department of Pediatrics, First Affiliated Hospital of Zhengzhou University, Zhengzhou, China; 2 Department of Neonatology, Children’s Hospital affiliated to Zhengzhou University, Henan Children’s Hospital, Zhengzhou Children’s Hospital, 33 Longhuwaihuan Road, Zhengzhou, 450018 China; 3 Department of Neonatology, Zhecheng People’s Hospital, Henan, China

**Keywords:** Retinopathy of prematurity, Preterm infant, Blood lactic acid, Impact, Level

## Abstract

**Background:**

Retinopathy of prematurity (ROP) is a common disease in premature infants. In recent years, most researchers have used lactic acid as poor prognosis marker in premature infants. This study aims to explore investigate the impact of blood lactic acid levels on ROP.

**Methods:**

A retrospective case-control study was conducted, and infants with severe ROP born with birth weight (BW) ≤ 1500 g and gestational age (GA) ≤ 32 weeks were enrolled from November 2016 to November 2021. Infants without any stage ROP were included as controls and were matched with ROP infants (1:2) by GA and BW. All selected preterm infants were tested for heel terminal trace blood gas analysis within two weeks of life. Changes in blood lactic acid levels in the two groups were compared and analyzed by using multivariate logistic regression analysis. Sensitivity and specificity were analyzed by receiver operating characteristic (ROC) curve.

**Results:**

There were 79 infants in ROP group, and 158 infants in control group. The levels of blood lactic acid were significantly higher in the ROP group on days 1, 3, 5, and 7 compared with control group (all *p* < 0.05). The blood lactic acid levels on day 5 was an independent risk factor for ROP (*p* = 0.017). The area under the curve (AUC), sensitivity and specificity were highest on day 5 (AUC 0.716, sensitivity 77.2% and specificity 62.0%, respectively, *p* < 0.001), and higher on days 1, 3, and 7.

**Conclusion:**

A high blood lactic acid level in the first seven days of life may be associated with increases ROP occurrence in very preterm infants, and suggest blood lactic acid level may impact the occurrence of ROP.

## Brief summary statement

Retinopathy of prematurity is a retinal vascular proliferative disease that endangers visual development in very preterm infants. This study investigated the impact of blood lactic acid levels on ROP by conducting a retrospective case-control study, and suggested relationship between lactic acid level and the occurrence of ROP.

## Introduction

Retinopathy of prematurity (ROP) is a retinal vascular proliferative disease that endangers visual and retinal development in very preterm infants [1]. ROP is the insufficiency of retinal blood vessels due to preterm birth, abnormal neonatal vascular proliferation, and fibroplasia in the retina without a vascular area [2]. It is a significant cause of childhood visual impairment and blindness worldwide [3]. A physiological hypoxic state was provided by normal intrauterine environment, which drives retinal angiogenesis and maturation of the retinal nerve sensory.[4] But abnormal intrauterine environment, such as chronic chorioamnionitis is associated with early-onset neonatal sepsis, metabolic acidosis and retinopathy of prematurity [5]. Metabolic acidosis is early risk factors for retinopathy of prematurity in preterm infants. The development of metabolic acidosis during chorioamnionitis is generally thought to be an increase in lactic acid due to proton release and sodium lactate formation [6, 7]. Several researchers have used blood lactic acid levels as a biomarker of poor prognosis in infants with very low or extremely low BW [8]. A previous study reported that the nucleated red blood cell count and serum lactic acid concentration are valuable biomarkers for predicting important outcomes in infants with very low birth weight [9]. 

Lactic acid is an intermediate product of in vivo glucose metabolism mainly produced by red blood cells, striated muscle, and brain tissues. Lactic acid is primarily produced under anaerobic conditions; tissue hypoxia can elevate lactic acid levels in the body. Hypoxia stimulates retinal vascular endothelial growth factor (VEGF) synthesis and neovascularization. High lactic acid levels can independently induce retinal VEGF generation depending on the lactic acid concentration [10]. A hypoxic environment increases retinal lactic acid levels seven-fold [11]. The lactic acid-induced VEGF expression mechanism alludes to its role in retinal neovascularization, suggesting that mechanisms other than hypoxia can stimulate retinal VEGF production, and that the combination of hyperlactic acid concentrations and hypoxia can further induce VEGF production [10]. 

The mechanism of lactic acid-induced VEGF expression is associated with the downregulation of adenosine diphosphate ribose. Adenosine diphosphate-ribosylation is a common post-translational protein modification. The high concentration of lactic acid forces lactic acid dehydrogenase to catalyze the conversion of nicotinamide adenine dinucleotide to reduced nicotinamide adenine dinucleotide. This effect of lactic acid on the conversion has resulted in speculations on its effect on the transcriptional rate and activity of VEGF [10]. Therefore, lactic acid values can provide information on cellular metabolic levels and reflect the actual oxygenation state of cells. Blood lactic acid levels have been shown to be easily detectable in the NICU through blood gas analysis. Arterial and venous lactic acid values are similar; therefore, blood lactic acid values can also be analyzed using heel tip trace blood gas analysis. The amount of data available on the predictive value of lactic acid in the association of lactic acid with morbidity and mortality in preterm infants is increasing [12]. Higher blood lactic acid levels were found during the blood gas analysis of preterm infants with pre-threshold or threshold disease ROP in clinical work. Therefore, it was speculated that blood lactic acid levels might influence ROP occurrence. This study aimed to explore the effect of blood lactic acid levels on ROP and its occurrence.

## Materials and methods

A retrospective case-control study was conducted between November 2016 and November 2021 at the Children’s Hospital affiliated to Zhengzhou University. The study has been approved by the Ethics Committee of the Children’s Hospital Affiliated to Zhengzhou University and was conducted in accordance with the provisions of the Declaration of Helsinki.

### Patient population

Neonates born with BW ≤ 1500 g and GA ≤ 32 weeks were enrolled in the study. Infants without any staged ROP were included as controls and matched with infants with ROP (1:2) by GA and BW. Infants with genetic metabolic diseases, congenital abnormalities, congenital heart disease, or infections were excluded.

### Data collection

Primary data were collected on the enrolled infants, including sex; GA; BW; blood gas analysis (including blood lactic acid); as well as treatment and related complications, including duration of mechanical ventilation; intracranial hemorrhage; necrotizing enterocolitis; sepsis; bronchopulmonary dysplasia; and blood transfusions.

### Screening and classification of ROP

ROP screening in the enrolled infants was performed by two experienced ophthalmologists using the Retcam III Wide Cape Digital Retina Imaging System (Massie, USA). ROP screening was performed and diagnosed as stages 1–5 according to the International Classification of ROP, [13] defined as follows: type 1 ROP, any stage ROP with “plus” disease, stage 3 ROP without plus disease in zone I, or stage 2 or 3 ROP with plus disease in zone II; and type 2 ROP, stage 1 or 2 ROP in zone I without plus disease, or stage 3 ROP in zone II without plus disease. Severe ROP was defined as more than stage 3 ROP and requirement of treatment.

### Blood lactic acid test

All selected preterm infants were tested for heel terminal trace blood (35 µl) gas analysis within two week of life, the frequency of blood gas analysis according to infant’s condition. For infants on ventilators, daily blood gas analysis, including pH, PCO_2_, PO_2_, and blood lactic acid, was tested. The test was performed using a fully automated blood gas analyzer (ABL90 FIEX, Denmark), and the blood gas analysis parameter including pH, PCO_2_, PO_2,_ and lactic acid level, and so on. We collected lactic acid from blood gas analysis data of days 1, 3, 5, 7, and 14.

### Statistical analysis

SPSS 22.0 was used to analyze the data. Quantitative data were expressed as mean ± standard deviation. Entry data and outcome differences were compared between two groups using t- test for normal distribution, and using non-parametric tests (Mann Whitney U test) for non-normal distribution. lactic acid in different times used ANOVA test. Count data were expressed as rates (%) and compared using chi-square tests. Potential confounders were adjusted by performing multiple logistic regression analyses. The sensitivity and specificity of blood lactic acid levels in ROP were analyzed by using ROC curve. Statistical significance was set at *p* < 0.05.

## Results

### Characteristics of baseline

**Fig. 1:**
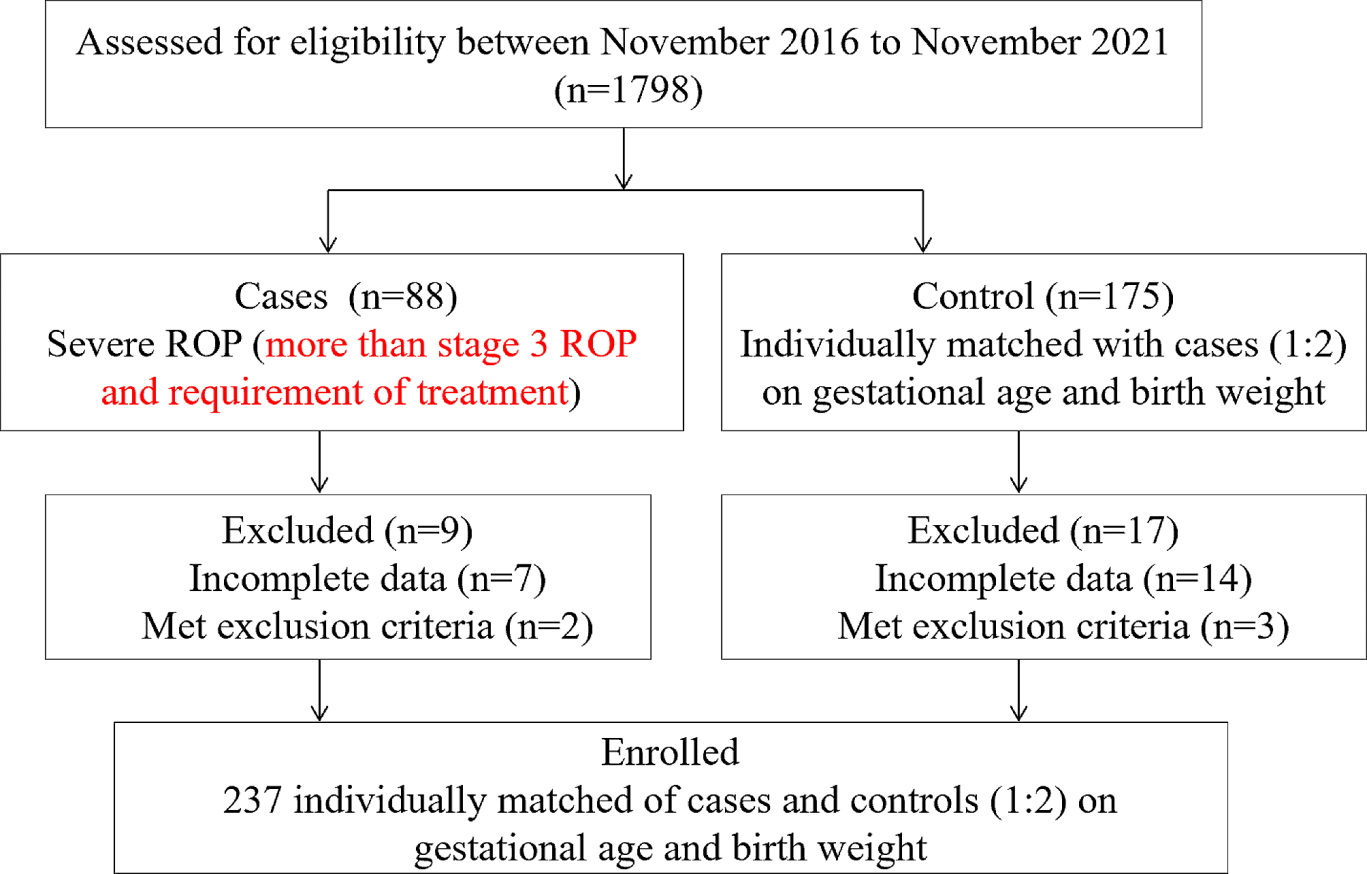
Flow chart describing the enrollment of the severe ROP cases and controls

1,798 preterm infants with GA ≤ 32 weeks and BW ≤ 1500 g were assessed for eligibility during the study period. Of these, 88 premature infants with severe retinopathy of prematurity excluded 9 infants because of incomplete data and meeting exclusion criteria, respectively. Finally, 79 infants with ROP were recruited, and 158 sex-matched infants with similar GA and BW without ROP were included in the control group (Fig. 1). There were 49 males and 30 females in the ROP group (GA, 24–32 weeks; BW, 500–1500 g). The control group consisted of 104 males and 54 females (GA, 24–32 weeks; BW, 510–1500 g). Baseline characteristics were similar in both groups beside of maternal chorioamnionitis and Apgar Scores at 5 min. There were higher rates of chorioamnionitis and lower Apgar Scores at 5 min in the ROP group compared to control group (*p* = 0.033 and *p* = 0.037, respectively). (Table 1).

**Table 1:** Baseline characteristics of all the infants enrolled in study

Characteristics	Control *n* = 158	ROP *n* = 79	P value
Male, n (%)	104(65.8)	49(62.0)	0.568
Gestational age(weeks), mean ± SD	29.9 ± 1.8	29.7 ± 1.9	0.429
Birth weight(gram), mean ± SD	1257 ± 253	1249 ± 249	0.818
Caesarean section, n(%)	95(60.1)	50(63.3)	0.673
Age(days), mean ± SD	51.3 ± 14.9	53.5 ± 15.7	0.293
Clinical chorioamnionitis, n (%)	10(6.3)	12(15.2)	0.033
Gestational diabetes, n (%)	6(4.0)	4(5.1)	0.735
Hypertension or pre-eclampsia, n (%)	15(9.5)	14(17.7)	0.091
Apgar Scores,5 min, mean ± SD	8.0 ± 1.8	7.5 ± 1.6	0.037
RDS	70(44.3)	33(41.8)	0.781

ROP, retinopathy of prematurity; RDS, respiratory distress syndrome

A comparison of clinical characteristics of the infants in the two groups revealed that days of mechanical ventilation and total parenteral nutrition were longer, and the number of transfusions was higher in the ROP group than in the control group (*p* = 0.004, *p* = 0.003, and *p* = 0.008, respectively). The incidence of sepsis, bronchopulmonary dysplasia, intracranial hemorrhage, and necrotizing enterocolitis was similar between the two groups. (Table 2).

**Table 2:** Clinical characteristics of infants in two groups

Characteristic	Control *n* = 158	ROP *n* = 79	P value
Sepsis, n (%)	26(16.5)	20(25.3)	0.118
NEC, n (%)	8(5.1)	5(6.3)	0.764
IVH, n (%)	24(15.2)	13(16.4)	0.850
BPD, n (%)	30(19.0)	22(28.2)	0.135
Days of Mechanical ventilation, mean ± SD	3.1 ± 5.3	5.2 ± 5.1	0.004
Number of transfusions, mean ± SD	1.1 ± 1.8	1.8 ± 2.1	0.008
TPN (days), mean ± SD	17.1 ± 7.5	20.9 ± 11.8	0.003

ROP, retinopathy of prematurity; BPD, bronchopulmonary dysplasia; IVH, intracranial hemorrhage; NEC, necrotizing enterocolitis; TPN, Total Parenteral Nutrition

### Outcomes of blood lactic acid levels

**Fig. 2:**
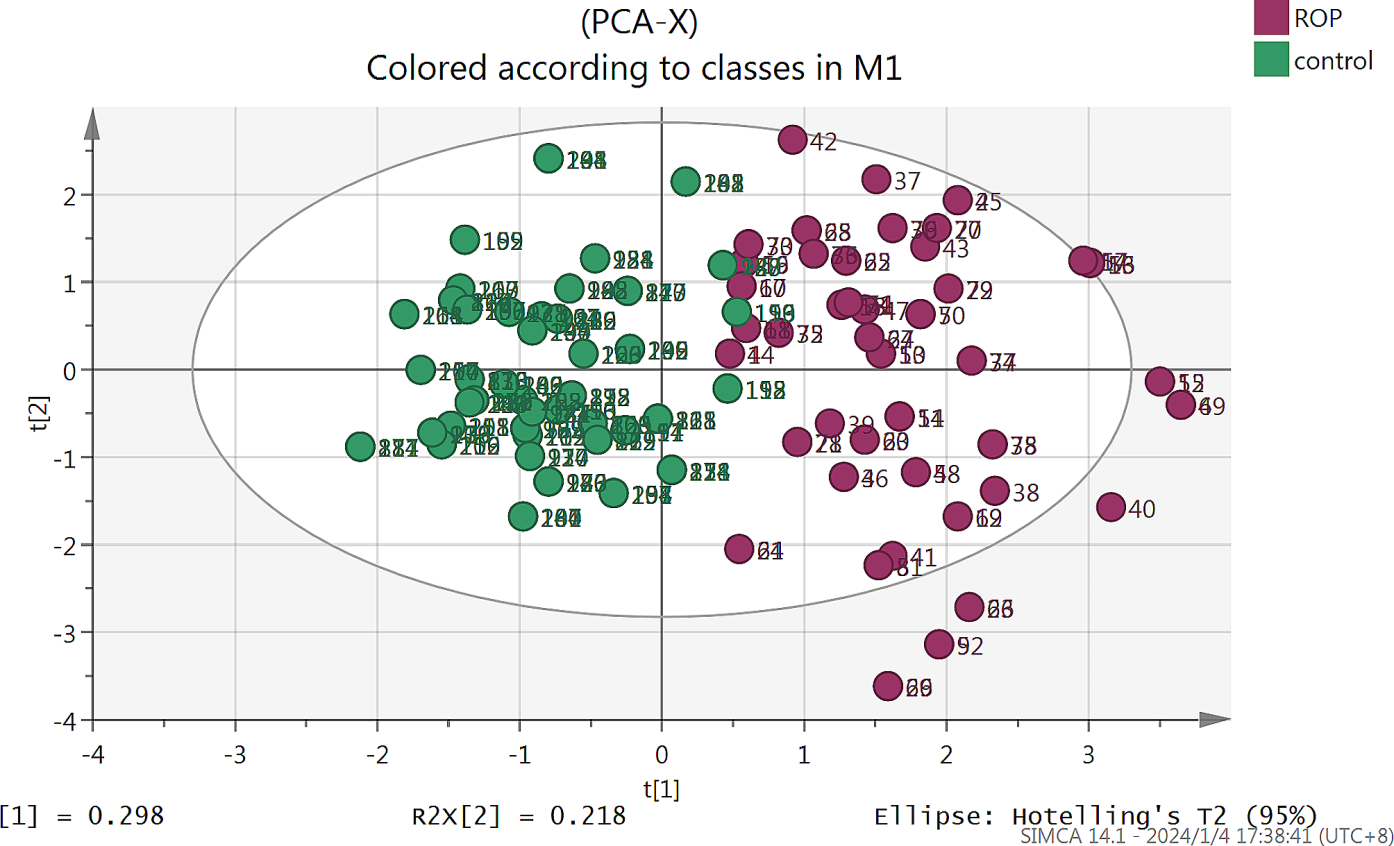
OPLS-DA score scatter plot offrst two principal components (ROP and control groups). Clear separate clustering can be observed between ROP and non- control groups

OPLS-DA score plot revealed a clear and separate clustering between premature infants with and without ROP (Fig. 2). This collectively suggest that this Model fits the data very well and has good predictive ability. There was a significant difference in the blood lactic acid levels between the two groups on days 1, 3, 5, and 7. In addition, blood lactic acid levels were higher in the ROP group than in the control group (all *p* < 0.05) (Table 3).

**Table 3:** Comparison of blood lactic acid between the two groups

	Control *n* = 158	ROP *n* = 79	P value
Blood lactic acid(mmol/L), mean ± SD			
Day 1	3.11 ± 1.74	4.96 ± 3.59	< 0.001
Day 3	2.55 ± 1.30	3.40 ± 2.22	< 0.001
Day 5	1.82 ± 0.82	2.64 ± 1.32	< 0.001
Day 7	1.49 ± 0.73	2.13 ± 1.51	< 0.001
Day 14	1.36 ± 0.51	1.40 ± 0.63	0.613

ROP, retinopathy of prematurity

Multivariate logistic regression analysis adjusted for potential confounding factors of ROP revealed that higher blood lactic acid levels last for 5 days increased the risk of ROP occurrence in the ROP group as compared to the control group. In addition, the model was adjusted for birth weight, maternal chorioamnionitis, Apgar scores at 5 min, the duration of mechanical ventilation and blood transfusions. The results showed that changes in blood lactic acid levels in the first seven days of life were associated with the occurrence of ROP (*p* < 0.05)(Table 4).

**Table 4:** multiple logistic regression analysis of the predict factors of blood lactic acid levels for severe ROP

	Unadjusted Standardized Coefficients				Adjusted Standardized Coefficients		
	Beta	Odds ratio (95% CI)	P value		Beta	Odds ratio (95% CI)	P value
Birth weight	-0.095	0.91(0.89–0.97)	0.003		-0.122	0.89(0.81–0.97)	0.008
Maternal chorioamnionitis	0.901	2.46(1.09–5.53)	0.029		0.565	0.57(0.15–2.14)	0.403
Apgar scores,5 min	-0.002	0.99(0.99–1.01)	0.590		-0.001	0.99(0.99–1.006)	0.696
Blood transfusion	0.306	1.36(0.82–2.26)	0.237		0.449	0.639(0.24–1.69)	0.366
Mechanical ventilation	0.062	1.06(1.00-1.13)	0.037		0.094	0.91(0.82–1.01)	0.072
blood lactic acid levels							
Day 1	0.278	1.32(1.17–1.49)	< 0.001		0.024	1.02(0.99–1.06)	0.156
Day 3	0.294	1.34(1.14–1.59)	0.001		0.035	1.04(0.99–1.07)	0.062
Day 5	0.764	2.15(1.61–2.86)	< 0.001		0.038	1.04(1.01–1.07)	0.017
Day 7	0.540	1.72(1.31–2.24)	< 0.001		0.028	1.03(0.99–1.07)	0.145

ROC curve analysis was used to analyze the sensitivity and specificity of blood lactic acid levels in the ROP group on varying postnatal days. The results showed that the area under the curve (AUC), sensitivity, and specificity peaked on day 7 (0.716, 77.2, and 62.0%, respectively; *p* < 0.001) and were lower on day 14. However, the difference was not statistically significant. The results are summarized in Table 5.

**Table 5:** Prediction of ROP by blood lactic acid levels in different postnatal days

age	AUC	standard error	sensitivity (%)	specificity (%)	P value	95% CI
Day 1	0.618	0.041	75.9	34.2	0.003	0.538 ~ 0.698
Day 3	0.588	0.043	68.4	36.7	0.027	0.504 ~ 0.672
Day 5	0.716	0.042	77.2	62.0	< 0.001	0.633 ~ 0.799
Day 7	0.585	0.043	70.9	41.8	0.033	0.502 ~ 0.669
Day 14	0.515	0.042	64.6	38.0	0.714	0.432 ~ 0.597

AUC, area under curve

## Discussion

Lactic acid has been used in many clinical studies as a predictor of patient morbidity and mortality. Clinical studies have reported a correlation between blood lactic acid levels and morbidity or mortality [14]. However, there is no consensus on the threshold of hyperlactic acidmia that should be used to predict complications during lactic acid elevation [15]. Lactic acid is produced in the hypoxic conditions and can be produced and metabolized by all tissues in the body. Information about metabolic capacity at the cellular level can be provided by lactic acid values, and true perfusion and oxygenation status were reflected [16]. Lactic acid is known to have multiple roles in cell homeostasis as a metabolic fuel and buffer, which acts as a signaling molecule called “Lactormone” and mediates this signaling through the hydroxy-carboxylic acid recptor 1 [17]. Hydroxy-carboxylic acid receptor 1 is a G-protein-coupled receptor activated by L-lactic acid and the exogenous agonist 3, 5-dihydroxybenzoic acid, also known as GPR81 [18]. It is expressed in a variety of organs including brain, retina, kidney, adipose tissue, liver rand skeletal muscle [19]. GPR81 has been found to mediate the effects of L-lactic acid in a variety of processes, including angiogenesis, wound healing, inhibition of inflammation, and neuroprotection [20]. This study found no significant difference in blood lactic acid levels between the ROP and control groups on day 14 of life. However, the infants in the ROP group had significantly higher postnatal blood lactic acid levels on days 1, 3, 5, and 7 of life than the controls (*p < 0.05*). Multivariate logistic regression analysis revealed that an elevated blood lactic acid level was a risk factor for the development of ROP, suggesting that increased mean blood lactic acid levels may impact the occurrence of ROP. However, no differences in lactic acid levels with respect to sex were found in this study. High lactic acid levels during the first few days of life reflect circulatory impairment and poor perfusion. Peripheral retinal hypoxia may contribute to worsening ROP [21]. Some reports indicated that neonatal asphyxia and acidosis in early of life were strongly associated with reduced IGF-1 levels [22]. With this knowledge, we can assume that the results of our study were the result of peripheral retinal hypoxia in conjunction with IGF-1 inhibition.

Retinopathy of prematurity is a vascular dysproliferative retinopathy that occurs in preterm infants. ROP is the major cause of visual loss and blindness in children, which is a multifactorial disease. The pathogenesis of ROP is divided into two stages: stage I, retinal vascular obstruction or blocked development; and stage II, neovascularization, followed by retinal hypoxia. In the normal intrauterine environment, the angiogenic stimulus is provided by VEGF beginning around 25 to 26 weeks gestation, with complete retinal vascularization by 40 weeks gestation [23]. 

Hyperoxic environment plays a role in the pathogenesis of ROP. However, For infants with the maintains of SaO2 was between 92–95% [24], Oxygen supplementation had no significant effects on the retina. However, a greater incidence of adverse pulmonary effects, including pneumonia and increased severity of chronic lung disease, was observed in the supplemented group. Thus, while it seems prudent to avoid chronic or episodic hypoxia in infants at risk for developing threshold ROP, the benefit of oxygen supplementation in excess of the minimum required to maintain tissue oxygenation and pulmonary homeostasis remains to be determined [25]. 

VEGF and its receptors play important roles in regulating neoangiogenesis. The rate of glycolysis is very high in the normal retina. Winkler et al. [26] reported that approximately 90% of glucose transfer in isolated rat retinas produced lactic acid under aerobic conditions and that the lactic acid concentration in the retina is higher than that in other tissues. The steady-state concentrations of glucose and lactic acid in the aqueous humor and vitreous fluid of the eye are reported to be 5 and 10 mM, respectively. [26] Under hypoxic conditions, glycolysis increases, and retinal circulation cannot remove excess lactic acid, resulting in a more significant accumulation of lactic acid in the retina.

Lactic acid, which plays an important role in retinal metabolism, is consumed mainly by photoreceptors supplied as a major fuel and coupled to the glutamate-glutamine cycle to power the interactions between photoreceptors and glia. Previous studies on retinal microvessels have shown that lactic acid can act as a vasoconstrictor when there is a sufficient energy supply, but as a vasodilator under hypoxic conditions intravenous administration of lactic acid can increase blood flow to the retina in rats. Injection of lactic acid into the vitreous of porcine eyes resulted in the dilation of retinal blood vessels > 70 μm in diameter [27]. In this study, the blood lactic acid levels were higher and oxygen saturation was lower in the first seven days of life in the ROP group. This result was similar to that of a previous study. Lactic acid is produced under anaerobic conditions and can be metabolized by all body tissues. Lactic acid values can provide information on metabolic capacity at the cellular level and reflect the true perfusion and oxygenation status [28]. A previous study reported that a single lactic acid value > 5.6 mmol/l had a sensitivity of 100% and specificity of 85% for identifying adverse outcomes. Persistently elevated or worsening lactic acid levels are associated with adverse outcomes [12]. In neonates admitted to a level-III NICU, arterial lactic acid levels within 3 h of birth were significantly higher in preterm neonates with poor neurodevelopmental outcome or death [29]. Serum lactic acid concentrations on day 5 of life are associated with retinopathy of prematurity, bronchopulmonary dysplasia, and intraventricular hemorrhage. Therefore, serum lactic acid concentration is a valuable biomarker for predicting important outcome parameters in infants born with very low BW [9].

Limitation in our study must be mentioned. At first, a retrospective study has many intrinsic limitations besides the measurements. Second, for infants with invasive and non-invasive ventilation or with high flow oxygen, we did twice blood gas to measure PaCO_2_, PaO_2_ (maintain 70–90 mmHg), and SaO_2_, and measure SpO_2_ using Philips Medizin systeme monitor. But for infants with nasal oxygen, we only measure SpO_2_ using Philips Medizin systeme monitor, and PaO2 was missed. Third, We only compared the outcomes for survives with severe ROP and not for the composite outcome of severe ROP or death in NICU, there may result in a selection bias.

the composite outcome of severe ROP or death in NICU. Only infants who survived to discharge may result in a selection bias.

Lactic acid plays an essential role in retinal metabolism. The results of this study suggest a correlation between blood lactic acid levels and the occurrence of severe ROP; a high blood lactic acid level in the first seven days of life may be associated with increases ROP occurrence in very preterm infants. Lactic acid may play a role in the development of ROP and is not just a biomarker. A further investigation of the relationship between blood lactic acid levels and ROP at the molecular biology level may be required.

## Data Availability

No datasets were generated or analysed during the current study.

## Abbreviations

ROP: retinopathy of prematurity
RR: risk rate
CI: confidence interval
SD: standard deviation
ROC: receiver operating characteristic curve
VEGF: vascular endothelial growth factor
PMA: postmenstrual age
VLBW: very low birth weight infants
NEC: necrotizing enterocolitis
BPD: bronchopulmonary dysplasia
ETROP: Early Treatment for Retinopathy of Prematurity
